# Unilateral low-load blood flow restriction vs. high-load training in the Bulgarian split squat: a randomized within-subject design on strength, hypertrophy, and asymmetry

**DOI:** 10.3389/fphys.2026.1786733

**Published:** 2026-03-30

**Authors:** Yanfei Wang, Xinyuan Zhao, Lingfeng Zhang, Xin Gao, Zhanfei Zheng, Shoudu Wang

**Affiliations:** 1 School of Physical Education, Chifeng University, Chifeng, Inner Mongolia, China; 2 School of Sports Training, Shenyang Sport University, Shenyang, Liaoning, China; 3 Department of Physical Education, Sichuan Electronic and Mechanic Vocational College, Mianyang, Sichuan, China; 4 Faculty of Sport, Catholic University of San Antonio of Murcia, Murcia, Spain; 5 Department of Sports Science, Wenzhou Medical University, Wenzhou, Zhejiang, China

**Keywords:** blood flow restriction training, Bulgarian split squat, high-load resistance training, muscle hypertrophy, unilateral training

## Abstract

**Purpose:**

To compare the effects of a 6-week unilateral low-load blood flow restriction (LL-BFR) training protocol versus traditional high-load resistance training (HL-RT) on maximal strength, muscle hypertrophy, and explosive power in resistance-trained men.

**Methods:**

Twenty-four resistance-trained males (age: 21.0 ± 2.0 years) completed a randomized within-subject trial. Each participant performed unilateral Bulgarian split squats (BSS) with one leg assigned to LL-BFR (30% 1RM, 30-15-15–15 repetitions at 80% arterial occlusion pressure [AOP]) and the contralateral leg to HL-RT (80% 1RM, 4 sets of 10 repetitions). Training consisted of 18 sessions over 6 weeks. Variable assessments, including BSS 1RM, vastus lateralis (VL) muscle thickness, and single-leg countermovement jump (SLCMJ) height and relative peak power were conducted both before (pretests) and after (posttests) the intervention.

**Results:**

Both conditions resulted in significant time-dependent improvements in BSS 1RM (LL-BFR: +10.7%; HL-RT: +13.0%), VL muscle thickness (LL-BFR: +8.1%; HL-RT: +9.3%), and SLCMJ performance (height: +10.7–13.0%; relative peak power: +6.5–8.7%) (all P < 0.05). Crucially, no significant condition × time interactions were detected for any variable, indicating that the magnitude of adaptation was comparable between the LL-BFR and HL protocols. Additionally, training did not exacerbate inter-limb asymmetry.

**Conclusions:**

Unilateral LL-BFR and HL-RT produced similar time-dependent improvements in maximal strength, muscle hypertrophy, and explosive performance in resistance-trained men. LL-BFR may be considered as a joint-sparing option when high mechanical loading is not feasible or desirable.

## 1 Introduction

Lower-limb strength and power are fundamental determinants of athletic performance, underpinning sprinting, jumping, change-of-direction ability, and reduced injury risk (Suchomel et al., 2018). High-load resistance training (HL-RT, ≥70% one-repetition maximum [1RM]) is widely regarded as the most effective strategy to maximize strength gains and is also highly effective for muscle hypertrophy (Schoenfeld et al., 2017). Systematic reviews comparing low- versus HL-RT with matched volume generally report greater improvements in maximal dynamic strength with heavier loads, although the magnitude of this advantage varies across populations, exercises, and outcomes (Lopez et al., 2021; Grgic et al., 2022). In parallel, unilateral multi-joint exercises such as the Bulgarian split squat (BSS) have gained prominence in strength and conditioning practice because they allow high relative loading with lower absolute loads, challenge lumbopelvic stability, and address sport-relevant asymmetries (Speirs et al., 2016). Recent work indicates that unilateral resistance training can produce adaptations with comparable transfer to unilateral strength, jump performance, and change-of-direction ability compared with bilateral training (Manca et al., 2017; Ramirez-Campillo et al., 2022). However, frequent exposure to heavy unilateral loading may be limited in athletes or physically active individuals with joint pain, congested competition schedules, or during rehabilitation phases.

Blood flow restriction (BFR) training has emerged as a promising strategy to induce robust neuromuscular adaptations while using substantially lower external loads (Patterson et al., 2019a). When pneumatic cuffs are applied proximally to partially restrict arterial inflow and markedly restrict venous return during low-load resistance exercise (typically 20–30% 1RM), meaningful increases in muscle size and strength can occur despite reduced mechanical stress (Loenneke et al., 2014). A growing body of randomized trials and meta-analyses indicates that low-load BFR (LL-BFR) produces hypertrophy comparable to HL-RT and superior to traditional low-load training without BFR, whereas strength gains are generally smaller than those achieved with HL-RT but clearly greater than low-load training alone (Lixandrao et al., 2018; Grønfeldt et al., 2020; Wortman et al., 2021). These findings have led expert panels to recommend LL-BFR as an option when high external loads are contraindicated, provided that cuff pressure, width, and application time are carefully individualized and monitored (Scott et al., 2023).

Despite this substantial evidence base, several important gaps remain. First, most LL-BFR versus HL-RT comparisons have used between-subject parallel designs in which one group performs LL-BFR and another group performs traditional HL-RT (Lixandrao et al., 2018; Centner et al., 2019a). Although such designs are practical, they are susceptible to inter-individual variability in genetics, training history, and lifestyle, which may obscure true differences between modalities. Second, the majority of BFR studies have focused on single-joint or machine-based exercises (e.g., knee extension or leg press), whereas less is known about LL-BFR applied to unilateral, closed-chain multi-joint tasks such as the BSS that more closely mirror sport-specific force production patterns and limb-specific loading (Su et al., 2025). Third, many studies have involved untrained or clinical populations, and fewer data are available in resistance-trained young adults, for whom adaptive ceilings and neural efficiency may modulate the relative effectiveness of LL-BFR versus HL-RT (Geng et al., 2024).

At the same time, unilateral resistance training raises questions about contralateral (cross-education) effects. Meta-analyses and mechanistic reviews show that training one limb can increase voluntary strength in the opposite, untrained limb by approximately 8–20%, largely via neural adaptations within supraspinal and spinal circuits (Green and Gabriel, 2018; Manca et al., 2018). This phenomenon is relevant to within-subject comparisons because systemic and neural responses may be partially shared across limbs, potentially attenuating differences between conditions. On the other hand, a unilateral within-subject model offers clear advantages. By exposing each participant to both LL-BFR and HL-RT in opposite limbs, between-subject variability is minimized and limb-specific adaptations can be compared under closely matched systemic conditions. Such designs have been used to contrast different loading intensities (20–80% 1RM) under volume-matched conditions, showing that higher intensities produce superior long-term strength gains despite similar hypertrophy (Appleby et al., 2020). To date, however, evidence directly contrasting LL-BFR and HL-RT within individuals using the same unilateral, closed-chain multi-joint exercise in a resistance-trained cohort remains limited. Therefore, the present study compared unilateral LL-BFR and HL-RT performed with the Bulgarian split squat in resistance-trained men, examining changes in muscle thickness, unilateral strength, jump performance, and inter-limb asymmetry. Based on prior evidence, we hypothesized that LL-BFR would induce hypertrophy comparable to HL-RT but result in smaller improvements in maximal strength.

## 2 Method

### 2.1 Participants

An *a priori* power analysis (α = 0.05, 1–β = 0.90) was performed using G*Power (Version 3.1.9.3) based on the primary outcome of BSS 1RM. The power calculation considered an ANOVA with repeated measures using a within-subject design, with two conditions (LL-BFR vs. HL-RT) and two time points (pre vs. post), focusing on the Condition × Time interaction. The correlation among repeated measures was conservatively set at 0.50 (ϵ = 1.0). The effect size was specified as Cohen’s f = 0.40 for the interaction term based on similar studies in the PAPE literature (Hu et al., 2025). To express our results with 95% confidence, a minimum sample size of 20 participants was obtained, with ten allowing for a 20% attrition rate. Therefore, twenty-four healthy males (age: 21.0 ± 1.98 years; height: 1.73 ± 0.06 m; body mass: 78.2 ± 9.3 kg; training experience: 3.15 ± 0.96) were recruited (**Supplementary Table 1**) (Guo et al., 2013). All participants were recruited from social media. Inclusion criteria were: male, 18–30 y, ≥2 years of resistance training (≥2 sessions·week^−1^), and back-squat 1RM ≥1.5× body mass (156.1 ± 20.1 kg). Exclusion criteria included: current lower-limb injury/pain, history of cardiovascular disease, and other contraindications to BFR. BFR contraindications were screened using a standardized health-history in line with methodological guidance for BFR exercise (Patterson et al., 2019b). A total of 30 screened participants were assessed, and 24 were enrolled and completed baseline testing.

Leg dominance was determined by self-report (preferred leg to kick a ball), a commonly used method with high agreement with observed dominance (Van Melick et al., 2017). Using a computer-generated randomization sequence, either the dominant or non-dominant limb was allocated to the LL-BFR condition, with the contralateral limb assigned to the HL-RT condition. Participants were screened for contraindications to BFR (e.g., cardiovascular disease, uncontrolled hypertension, thrombotic events, or other relevant medical conditions) and provided written informed consent prior to participation. The study was approved by the *Institutional Review Board of Wenzhou Medical University (no. 2025082)* and conducted in accordance with the Declaration of Helsinki. Participants were instructed to maintain habitual diet and physical activity, refrain from additional lower-body resistance training during the intervention, avoid strenuous exercise for 48 h, and abstain from alcohol and caffeine for 24 h before each testing session. Compliance with these instructions was monitored by standardized verbal confirmation before each testing session and during supervised training visits.

### 2.2 Study design

This study used a randomized within-subject design in which each participant trained both lower limbs concurrently under two different loading conditions: (A) low-load resistance exercise with blood flow restriction (LL-BFR) in one limb and (B) traditional HL-RT in the contralateral limb. A computer-generated randomization sequence assigned LL-BFR to either the dominant or non-dominant limb. Order of training between limbs was randomized and counterbalanced across sessions. After screening and recruitment, participants attended one familiarization session during which they were instructed in the standardized warm-up, Bulgarian split squat (BSS) technique, single-leg jump testing procedures, and the sensations associated with LL-BFR exercise. Within 3–7 days after familiarization, baseline measurements were obtained for both legs, including unilateral BSS-1RM, muscle thickness, and single-leg countermovement jump (SLCMJ) performance. Outcome assessors for strength and jump testing, as well as ultrasound acquisition/analysis, were blinded to limb allocation. Participants and training supervisors were not blinded due to the nature of BFR. Participants then completed a 6-week training intervention in which both limbs performed unilateral BSS under different loading conditions consisting of three supervised sessions per week (18 sessions in total). All training sessions were conducted in the same facility, supervised by experienced strength and conditioning professionals, and session attendance was recorded to monitor adherence. Post-intervention testing was conducted using the same protocol as the baseline testing, as described above.

### 2.3 Procedures

#### 2.3.1 Training intervention

Participants completed 18 training sessions over a 6-week period (three sessions per week). All sessions were supervised by experienced strength and conditioning professionals. Each session began with a standardized warm-up consisting of 5 min of stationary cycling (Wattbike, West Bridgford, United Kingdom; 60 rpm, 60 W) followed by dynamic lower-limb stretches and submaximal bodyweight squats. For the LL-BFR limb, the external load was set at 30% of the unilateral BSS 1RM determined at baseline. Participants performed 4 sets of 30–15–15–15 repetitions (1 s concentric; 2 s eccentric) with 30 s passive rest between sets. This exercise protocol has been widely used in studies examining the physiological responses to BFR resistance exercise (Loenneke et al., 2013; Mendonca et al., 2021). Blood flow restriction was applied using an automatic pneumatic system (KAATSU B2 wearable, KAATSU, Tokyo, Japan) with 13-cm-wide thigh cuffs. Arterial occlusion pressure (AOP) was estimated individually using the KAATSU B2 manufacturer procedure based on thigh circumference (measured at the standardized site) and cuff characteristics, and training pressure was set at 80% of the predicted AOP (253.3 ± 18.9 mmHg) (Loenneke et al., 2012a; Murray et al., 2021). AOP estimation was performed with participants in the standing position. Thigh circumference was re-assessed at week 3 and cuff pressures were updated. The cuff was inflated immediately before each set and fully deflated immediately after the last repetition of each set, remaining deflated throughout the inter-set rest period. The contralateral limb allocated to the HL-RT condition trained at 80% of its BSS 1RM without BFR. HL-RT consisted of 4 sets of 10 repetitions (1 s concentric; 2 s eccentric) of BSS at 80% 1RM, and 120 s of passive inter-set recovery (American College of Sports M, 2009; Speirs et al., 2016). Participants were instructed to terminate a set if they could no longer complete a repetition with proper technique within the prescribed repetition range. Loads were prescribed relative to baseline 1RM (HL-RT: 80%; LL-BFR: 30%). Sets were terminated if technique could not be maintained within the prescribed repetition scheme. No formal effort scale was collected, and session-by-session external loads were not systematically recorded.

### 2.3.2 Anthropometrics

Stature was measured barefoot to the nearest 0.1 cm using a wall-mounted stadiometer (Seca 213, Hamburg, Germany). Body mass was measured to the nearest 0.1 kg using a calibrated digital scale (Seca 769, Hamburg, Germany). Participants stood upright with feet shoulder-width apart and weight evenly distributed on both legs, with the muscles relaxed. The measurement site was located at 1/3 of the distance between the inguinal crease and the proximal border of the patella on the anterior aspect of the thigh and marked with semi-permanent ink to ensure consistency across sessions (Zhang et al., 2024). Two measurements were taken per leg; if they differed by > 0.5 cm, a third was obtained and the median recorded. All assessments were performed at the same time of day by the same trained examiner.

#### 2.3.3 Unilateral Bulgarian split squat one-repetition maximum

Maximal strength of each lower limb was assessed using a unilateral Bulgarian split squat 1RM test performed on a Smith machine (Andersen et al., 2014; Chen et al., 2025). After a standardized warm-up (5 min cycling, dynamic stretches, and specific BSS warm-up sets), participants performed single-leg attempts with the rear foot supported on a 40-cm bench (Helme et al., 2019). Standardized depth was defined as the anterior thigh of the front leg reaching parallel to the floor, monitored via visual observation and verbal feedback. Loads began at ~60–70% estimated 1RM and increased by 2.5–5.0 kg per successful attempt until failure, with 3–5 min rest between attempts. A successful lift required completion of the full range of motion without assistance, excessive compensation, or loss of balance. Each limb was tested separately. Limb order was counterbalanced, and ≤5 maximal attempts per limb were allowed. The heaviest successful load was recorded as 1RM (kg).

#### 2.3.4 Muscle thickness

VL muscle thickness was assessed bilaterally using B-mode ultrasound (LOGIQ S8, GE Healthcare, Milwaukee, WI, USA) following standard procedures. Participants rested supine for 10 min in a temperature-controlled laboratory prior to imaging. Measurement sites were marked at 50% of the distance between the greater trochanter and lateral femoral epicondyle. A linear-array transducer (7.5–12 MHz) was positioned perpendicular to the tissue with ample coupling gel and light contact to avoid visible tissue compression. Images were saved and analyzed off-line (three longitudinal images per leg), and the average MT was recorded. All scanning and analysis were performed by a single, experienced examiner blinded to limb allocation, with limbs coded (e.g., Leg A/Leg B) during acquisition and analysis to maintain blinding. Test-retest reliability was excellent (ICC > 0.90; CV < 5%).

#### 2.3.5 Single-leg countermovement jump

Unilateral explosive performance was assessed using the (SLCMJ on a force platform (Kistler 9286BA, Winterthur, Switzerland). After a specific warm-up, participants performed 1–2 submaximal practice trials per leg, followed by a maximum of five maximal SLCMJ trials per leg. Participants stood on the test limb with hands on hips, performed a self-selected countermovement, and jumped vertically as high as possible, landing on the same limb. Trials were discarded and repeated if balance was lost, the contralateral limb provided support, hands left the hips, or a clear pause occurred at the bottom of the countermovement. A 60-s rest interval was provided between trials. Jump height and relative peak power output (peak power normalized to body mass; W·kg^−1^) were all calculated using the MARS software (version 4.0; Kistler Corporation, Switzerland). Within-session reliability was excellent (ICC > 0.90; CV < 5%).

#### 2.3.6 Inter-limb asymmetry indices

To quantify limb-to-limb discrepancies, inter-limb asymmetry indices were calculated for the primary unilateral outcomes (SLCMJ height, SLCMJ peak power output, and BSS 1RM). At each time point, asymmetry was expressed as:


$$Asymmetry\;(\%)=\frac{\left|Higher\;limb-Lower\;limb\right|}{Higher\;limb}\times 100$$

This calculation provides a non-directional asymmetry score, with higher values indicating greater inter-limb disparity. Pre-to-post changes in asymmetry were used to examine whether inter-limb discrepancies were altered following the intervention.

### 2.4 Statistical analysis

Data are presented as mean ± SD. A two-way repeated-measures ANOVA with two within-subject factors (Condition [HL-RT and LL-BFR]) × Time (pre, post) was used to analyze each dependent variable (BSS 1RM, VL thickness, SLCMJ height, and SLCMJ peak power). The primary inference was the Condition × Time interaction. To account for testing across the four pre-specified primary outcomes, interaction p-values were adjusted using the Holm–Bonferroni procedure (family-wise α = 0.05). Assumptions were assessed for each model by inspection of residuals (Q–Q plots) and the Shapiro–Wilk test. Sphericity was not applicable because each factor had two levels. When significant main effects or interactions were observed, Bonferroni-adjusted *post hoc* comparisons were performed. Inter-limb asymmetry indices were computed as a single non-directional score per participant at each time point and therefore compared between pre- and post-intervention using paired t-tests. Effect sizes for ANOVA were quantified using partial eta-squared (η_p_²), interpreted as small (≥0.01), moderate (≥0.06), and large (≥0.14). Cohen’s d was calculated and interpreted as trivial (<0.2), small (0.2–0.5), moderate (0.5–0.8), and large (>0.8). All analyses were performed in SPSS (v27.0; IBM Corp., Armonk, NY, USA), with statistical significance set at p ≤ 0.05.

## 3 Results

All participants completed all 18 supervised sessions (100% adherence; 0 dropouts). Adverse events/side effects were not systematically recorded; no session required medical assistance or discontinuation due to symptoms. The main effect of time was observed for SLCMJ height (F(1,23) =86.15) and peak power output (F(1,23) =86.18), BSS 1RM (F(1,23) =196.10), and vastus lateralis thickness (F(1,23) =72.59) (all P ≤ 0.001). No main effect of condition was found (all P ≥ 0.161), and no significant condition × time interaction was detected for any variable (all P ≥ 0.077). The smallest condition × time interaction P-value was observed for BSS 1RM (P = 0.077; ηp² = 0.130).

**Figure 1:**
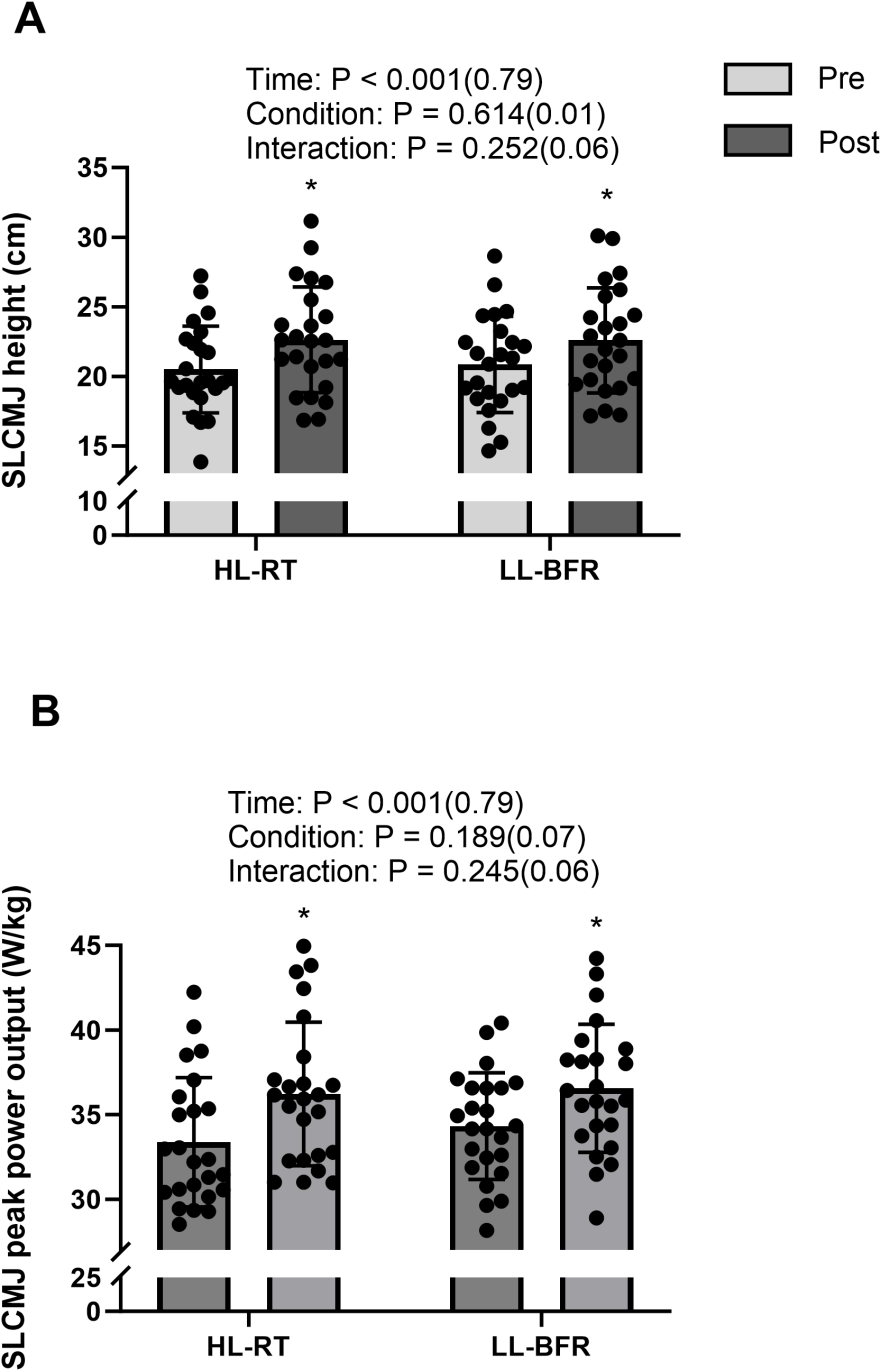
— SLCMJ height **(A)** and peak power output **(B)** before (pretests) and after (posttests) the 6-week intervention. SLCMJ indicates Single-leg countermovement jump; HL-RT, High-load resistance training; LL-BFR, low-load blood-flow-restriction. *Significant difference from pretests (P < 0.05).

**Figure 2:**
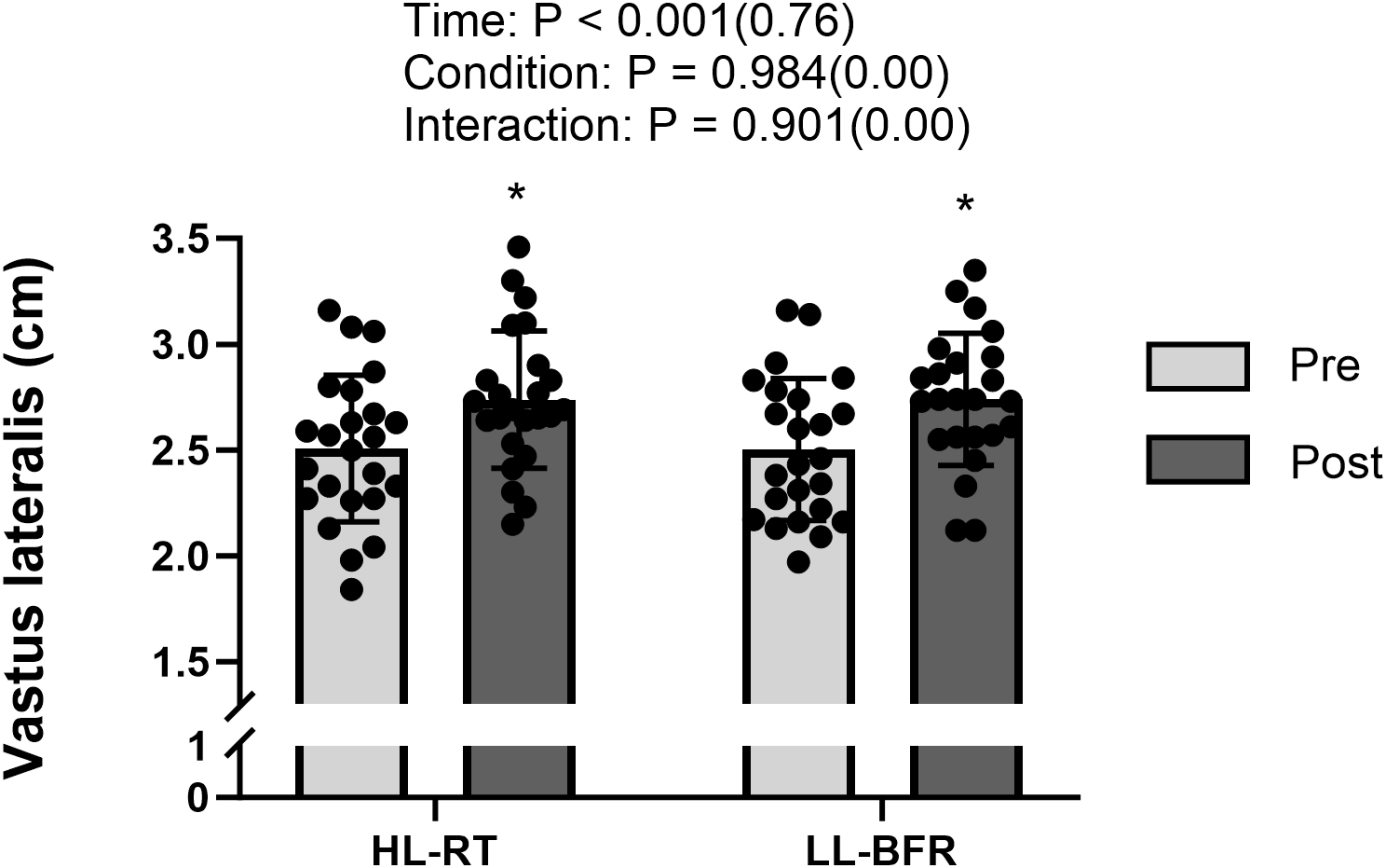
— Vastus lateralis thickness before (pretests) and after (posttests) the 6-week intervention. HL-RT, High-load resistance training; LL-BFR, low-load blood-flow-restriction. *Significant difference from pretests (P < 0.05).

**Figure 3:**
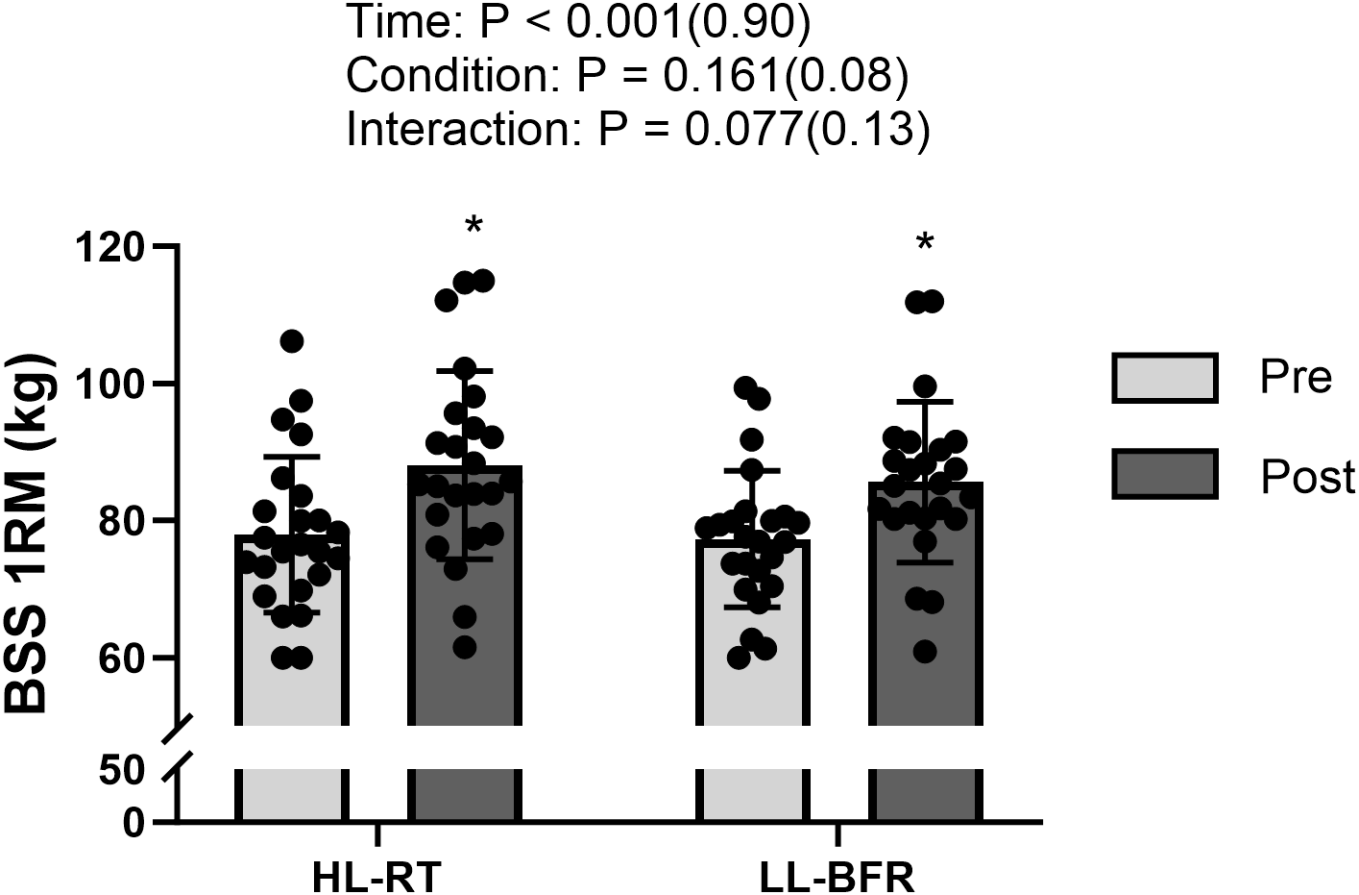
— BSS 1RM before (pretests) and after (posttests) the 6-week intervention. HL-RT, High-load resistance training; LL-BFR, low-load blood-flow-restriction. BSS: Bulgarian split squats. *Significant difference from pretests (P < 0.05).

Participants exhibited increased SLCMJ height in both HL-RT (20.5 ± 3.1 vs 22.6 ± 3.8 cm; 13.0% ± 5.4%; P < 0.001; d = 1.54) and LL-BFR (20.9 ± 3.5 vs 22.6 ± 3.8 cm; 10.7 ± 4.2%; P < 0.001; d = 1.38) from pretests to posttests (**Figure 1A**). The mean change was 2.13 cm (95% CI: 1.55 to 2.72) for HL-RT and 1.73 cm (95% CI: 1.20 to 2.26) for LL-BFR, with a between-condition difference in change (ΔHL−ΔLL) of 0.40 cm (95% CI: −0.31 to 1.11). Compared with pretests, SLCMJ peak power output increased after HL-RT (33.4 ± 3.8 vs 36.2 ± 4.2 W/kg; 8.7 ± 7.0%; P < 0.001; d = 1.35) and LL-BFR (34.3 ± 3.1 vs 36.6 ± 3.8 W/kg; 6.5% ± 4.5%; P < 0.001; d = 1.44) (**Figure 1B**). The mean change was 2.86 W/kg (95% CI: 1.96 to 3.76) for HL-RT and 2.23 W/kg (95% CI: 1.58 to 2.89) for LL-BFR, with ΔHL−ΔLL of 0.63 W/kg (95% CI: −0.46 to 1.71). Both HL-RT (2.51 ± 0.35 vs 2.74 ± 0.33 cm; 9.8 ± 7.6%; P < 0.001; d = 1.41) and LL-BFR (2.50 ± 0.34 vs 2.74 ± 0.31 cm; 10.0 ± 8.2%; P < 0.001; d = 1.26) resulted in vastus lateralis thickness from pretests to posttests (**Figure 2**). The mean change was 0.23 cm (95% CI: 0.16 to 0.30) for HL-RT and 0.24 cm (95% CI: 0.16 to 0.32) for LL-BFR, with ΔHL−ΔLL of −0.01 cm (95% CI: −0.10 to 0.09). From pretests to posttests, BSS 1RM improved for both HL-RT (77.9 ± 11.4 vs 88.1 ± 13.7 kg; 13.0 ± 5.4%; P < 0.001; d = 2.23) and LL-BFR (77.3 ± 10.0 vs 85.6 ± 11.7 kg; 10.7 ± 4.2%; P < 0.001; d = 2.40) (**Figure 3**). The mean change was 10.18 kg (95% CI: 8.25 to 12.11) for HL-RT and 8.32 kg (95% CI: 6.86 to 9.79) for LL-BFR, with ΔHL−ΔLL of 1.85 kg (95% CI: −0.21 to 3.92). Finally, inter-limb asymmetry did not change significantly from pre- to post-intervention for any variable (all P ≥ 0.32). Specifically, SLCMJ height asymmetry was 5.8 ± 4.3% at pretest and 6.1 ± 5.4% at posttest (P = 0.84; d = 0.04), with a mean change of 0.28% (95% CI: −2.61 to 3.18). SLCMJ peak power asymmetry was 6.3 ± 3.6% and 6.9 ± 3.5%, respectively (P = 0.56; d = 0.12), with a mean change of 0.57% (95% CI: −1.40 to 2.53). BSS 1RM asymmetry was 4.7 ± 3.7% at pretest and 5.6 ± 4.3% at posttest (P = 0.32; d = 0.21), with a mean change of 0.90% (95% CI: −0.93 to 2.73) (**Table 1**).

**Table 1:** Inter-limb asymmetry (%) before and after the 6-week intervention.

Variable	Pretests, mean ± SD (%)	Posttests, mean ± SD (%)	P value	Cohen's d
SLCMJ height	5.8 ± 4.3	6.1 ± 5.4	0.84	0.04
SLCMJ peak power output	6.3 ± 3.6	6.9 ± 3.5	0.56	0.12
BSS 1RM	4.7 ± 3.7	5.6 ± 4.3	0.32	0.21

Data are presented as mean ± standard deviation (SD). SLCMJ, Single-leg countermovement jump; BSS, Bulgarian split squats; P values and Cohen’s d refer to pre–post paired comparisons.

For each outcome, 95% CIs for within-condition changes (Post − Pre), post-test between-condition differences (HL-RT − LL-BFR), and the between-condition difference in change (Δdiff) are provided in **Supplementary Table 2**. Standardized interaction effect sizes (Hedges’ g for Δdiff) with 95% CIs are visualized in **Supplementary Figure 1**.

## 4 Discussion

### 4.1 Main findings

This study aimed to compare the effects of 6 weeks of unilateral Bulgarian split squat training performed as traditional HL-RT (~80% 1RM) or low-load blood-flow-restriction training (LL-BFR; 30% 1RM at ~80% AOP) on lower-limb adaptations in resistance-trained men. The main findings were that both HL-RT and LL-BFR produced large and statistically significant improvements in Bulgarian split squat 1RM (~11–13%), vastus lateralis muscle thickness (~10%), and single-leg countermovement-jump height and relative peak power (~7–13%). Importantly, no significant Condition × Time interactions were observed for any variable. In addition, inter-limb asymmetry in strength did not significantly change after the intervention. Collectively, these results suggest similar time-dependent improvements following both protocols within this concurrent unilateral model.

### 4.2 Strength and hypertrophy adaptations

Consistent with the changes observed in muscle morphology, our study demonstrated that performance gains for 1RM Bulgarian split squat (BSS) were not significantly different between LL-BFR and HL-RT conditions. Specifically, both conditions elicited substantial improvements in unilateral lower-limb strength (LL-BFR: +13.2%; HL-RT: +14.1%) following the 6-week intervention. These findings align with the meta-analysis by Grønfeldt et al (Grønfeldt et al., 2020), who reported that low-load blood flow restriction training (LL-BFR) can induce maximal strength gains of a similar magnitude to HL-RT in healthy populations, provided the training is conducted to volitional failure or with high metabolic stress. Similarly, Lixandrao et al. (2018) observed that while HL-RT generally favors 1RM improvements due to mechanical specificity, LL-BFR (20-40% 1RM) remains a potent stimulus for strength development, potentially initiating neural adaptations comparable to heavier loads. However, our results contrast with those of Cook et al. (2014), who reported that HL-RT was superior to LL-BFR for improving 1RM strength in semi-professional athletes. Additionally, a systematic review by Centner et al. (2019b) suggested that while trained individuals benefit from BFR, the magnitude of strength gain is often maximized with higher mechanical tension (i.e., HL-RT). Discrepancies in findings likely stem from the specific nature of the exercise modality tested (e.g., the high stability of the bilateral leg press in Cook et al. (2014) vs. the high neuromuscular demand of the unilateral BSS in this study) and the characteristics of the population, which can influence neuromuscular demands and transfer to 1RM outcomes. Our subjects possessed a high baseline strength level (back squat 1RM ~2.0× body mass). It is plausible that for such advanced individuals performing complex unilateral movements, the systemic and local metabolic stress induced by LL-BFR provided a novel stimulus sufficient to drive neural drive and motor unit recruitment patterns similar to those of HL-RT, thereby masking the typical superiority of heavy loads for 1RM strength.

Regarding structural adaptations, we observed significant and similar increases in Vastus Lateralis muscle thickness for both conditions (LL-BFR: +8.1%; HL-RT: +9.3%). This is consistent with the broad consensus in the literature, which posits that LL-BFR and HL-RT are similarly effective for inducing muscle hypertrophy when sets are performed to failure (Slysz et al., 2016; Lixandrao et al., 2018). For instance, Loenneke et al. (2012b) demonstrated that despite the lower mechanical tension in BFR protocols, the accumulation of metabolites (e.g., lactate, H+) and the subsequent cell swelling and growth factor release (e.g., GH, IGF-1) stimulate muscle protein synthesis pathways (mTORC1) to a degree comparable to HL-RT. However, discrepancies exist in the time course of these adaptations. Some studies suggest that BFR-induced hypertrophy may occur earlier (e.g., within 4 weeks) due to acute cell swelling effects, whereas HL-RT relies more on myofibrillar protein accretion over longer periods (Scott et al., 2016). While our 6-week timeframe limits the ability to distinguish these temporal nuances, LL-BFR may be considered to promote hypertrophy when high mechanical loading is not feasible or desirable, within the context of the present model. The comparable hypertrophy observed in our study, despite the vast difference in external load (approx. 30% vs. 75% 1RM), supports the application of Henneman’s size principle suggesting that the hypoxic environment of BFR forces the premature recruitment of high-threshold type II muscle fibers, mimicking the recruitment patterns typically reserved for HL-RT (Henneman et al., 1965).

### 4.3 Explosive performance and jump adaptations

Consistent with the improvements observed in maximal strength, our study demonstrated that SLCMJ height increased significantly in both the LL-BFR and HL-RT (+6.2% and +6.9%, respectively), with no significant differences between the training modalities. These findings align with the work of Cook et al. (2014), who observed that a 3-week occlusion training intervention in semi-professional rugby players resulted in significant improvements in countermovement jump peak power and 40m sprint times, suggesting that BFR can effectively enhance explosive characteristics in trained populations. Similarly, Abe et al. (2006) reported that LL-BFR training (20% 1RM) induced improvements in standing long jump distance and sprint speed comparable to traditional resistance training, attributing these gains to the preferential recruitment of fast-twitch (Type II) muscle fibers necessitated by the hypoxic environment. However, our results contrast with the velocity-specificity principle highlighted by Cormie et al. (2011), which suggests that training at slow velocities—typical of the fatigue-induced repetitions in BFR—should theoretically result in inferior adaptations for high-velocity tasks (e.g., jumping) compared to heavy resistance training or power-oriented protocols. Furthermore, a study by Fatouros et al. (2000) indicated that while varying loads can improve vertical jump, heavy loads or plyometrics typically yield superior transfer to explosive performance compared to low-load protocols lacking an explosive intent. Discrepancies in findings likely stem from the specific nature of the exercise selection (unilateral BSS mimicking the mechanics of the SLCMJ) and the physiological mechanisms of fatigue. In the present study, the specific biomechanics of the Bulgarian split squat—requiring unilateral hip and knee extension with stabilization—share a high degree of dynamic correspondence with the single-leg jump test. Although the movement velocity in the LL-BFR group may have decreased towards the end of sets due to metabolic accumulation, the “intent” to move the load ballistically likely remained high. This maximal voluntary effort under fatigue has been shown to result in high rates of motor unit firing and the recruitment of high-threshold motor units, which are critical determinants of explosive power (Pearson and Hussain, 2015). These SLCMJ improvements may reflect neural adaptations accompanying strength gains (e.g., improved motor unit recruitment/rate coding), in addition to morphological changes. Thus, despite the lower external load, the LL-BFR condition may have provided a sufficient neural stimulus to enhance the rate of force development (RFD) required for jumping, leading to performance gains similar to those of the HL-RT group.

### 4.4 Inter-limb asymmetry and cross-education

A notable finding of our study was the normalization of limb symmetry observed following the 6-week intervention. Initially, participants exhibited varying degrees of strength asymmetry; however, both LL-BFR and HL-RT protocols were effective in maintaining or improving the Limb Symmetry Index (LSI) for 1RM BSS, with no significant differences between conditions. Consistent with these results, Gonzalo-Skok et al. (2017) reported that unilateral resistance training (e.g., unilateral squat and lunge) was superior to bilateral training in reducing between-limb force asymmetries in team-sport athletes. They attributed this to the fact that unilateral exercises prevent the “compensation mechanism” often seen in bilateral movements, where the stronger limb masks the deficits of the weaker one. In contrast, Bishop et al. (2018) noted that while asymmetry is prevalent in athletes, not all training interventions successfully mitigate it, particularly if the training stimulus does not specifically target the weaker limb’s neural deficits. The successful management of asymmetry in our study suggests that the unilateral nature of the BSS exercise itself may be the primary driver for symmetry adjustments, rather than the magnitude of the external load.

Given the within-subject design employed in this study, the potential role of cross-education warrants discussion. We observed substantial strength gains in both limbs, which raises the possibility that the high neural drive generated by the HL-RT limb may have transferred to the LL-BFR limb (or vice versa). This is consistent with the meta-analysis by Manca et al. (2017), which indicates that unilateral resistance training can induce an average strength gain of approximately 11.9% in the contralateral limb, primarily due to neural spillover and cortical adaptations. However, Madarame et al. (2008) found that while cross-transfer exists, the addition of BFR to the “trained” limb did not significantly enhance the cross-education effect on the “untrained” limb compared to normal low-load training. Our findings diverge slightly from typical cross-education studies because both limbs were trained concurrently (albeit with different loads). It is plausible that the strength gains in the LL-BFR leg were not solely due to local adaptations but were augmented by a central neural “priming” effect from the heavy loading of the HL-RT leg. Discrepancies between our findings and strictly unilateral studies (where one leg rests) likely stem from the bilateral training volume and the systemic hormonal environment created when both large muscle groups are exercised in the same session. As suggested by Beyer et al. (2016), the systemic release of anabolic hormones and the global reduction in antagonist co-activation might blur the distinct physiological boundaries between the two loading protocols in a split-body design. Importantly, cross-education would be expected to bias the within-subject comparison toward smaller between-condition differences, thereby increasing the likelihood of observing a non-significant Condition × Time interaction even if small true differences exist. Therefore, the present findings should be interpreted as “no evidence of a differential effect within this design” rather than evidence of equivalence. Future studies using a parallel-group design or including an untrained control limb, and/or incorporating neurophysiological measures, are needed to better quantify and isolate cross-education effects.

## 5 Practical applications

Unilateral lower-limb training is critical for correcting asymmetries and enhancing athletic performance. However, the application of HL-RT in complex movements like the Bulgarian split squat may be constrained by axial loading, balance requirements, and cumulative joint stress, particularly during in-season microcycles or early-stage rehabilitation. Within this concurrent unilateral model, LL-BFR produced similar time-dependent improvements in strength and muscle thickness while using substantially lower external loads. Therefore, practitioners may consider LL-BFR as a joint-sparing strategy when high mechanical loading is not feasible or desirable, while recognizing that conclusions should be interpreted within the constraints of the present design.

## 6 Strengths and limitations

Several methodological factors and constraints should be considered when interpreting our findings. Firstly, the relatively brief 6-week intervention period may not have been sufficient to fully capture the potential long-term divergence in maximal strength gains, as HL-RT is often hypothesized to elicit greater neural-driven adaptations than LL-BFR over extended durations. Secondly, while our assessment included muscle thickness, the study lacks neuromechanical data (e.g., surface electromyography) and molecular analysis (e.g., muscle fiber type-specific hypertrophy), thus preventing a definitive mechanistic explanation for the comparable adaptations observed. Thirdly, our findings are specific to the unilateral Bulgarian split squat exercise and were obtained from a highly specific cohort of well-trained young men with high baseline strength, limiting the direct extrapolation of these results to bilateral movements or other populations such as females, older adults, or sedentary individuals. Finally, cross-education effects could not be quantified or controlled in this concurrent within-subject design and may have attenuated between-condition differences. Notwithstanding these limitations, the study benefits from a randomized controlled design and high ecological validity through the use of the BSS exercise, providing a novel benchmark for comparing the efficacy of low-load, high-metabolic-stress training (LL-BFR) against high-load mechanical tension (HL-RT) in a highly resistance-trained population.

## 7 Conclusion

Both protocols significantly improved vastus lateralis thickness, unilateral 1RM strength, and SLCMJ performance, with no significant changes in inter-limb asymmetry and no significant Condition × Time interactions. Within this concurrent unilateral model, LL-BFR may be considered when high mechanical loading is not feasible or desirable. Future studies should test longer interventions and broader populations. Future studies should examine longer interventions and broader populations to clarify whether small between-condition differences emerge over time.

## Data Availability

The raw data supporting the conclusions of this article will be made available by the authors, without undue reservation.
