# Supplementary figures and images for: Unilateral low-load blood flow restriction vs. high-load training in the Bulgarian split squat: a randomized within-subject design on strength, hypertrophy, and asymmetry
Source: Front Physiol. 2026 Mar 30;17:1786733. doi: 10.3389/fphys.2026.1786733 (PMC13070802; doi:10.3389/fphys.2026.1786733)

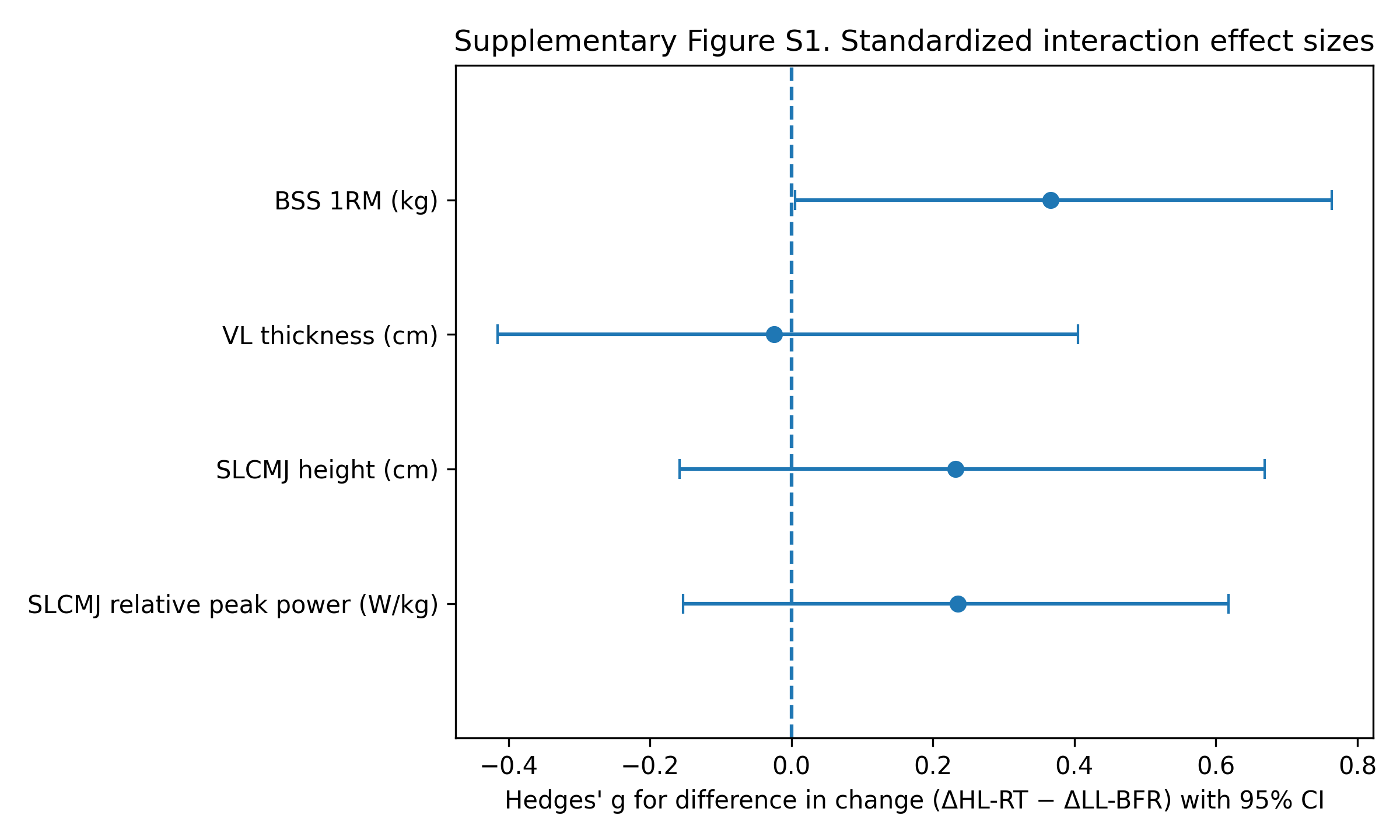

Supplement: Supplementary file 1 [file Image1.png]
